# Supplementary material for: PAL-AI reveals genetic determinants that control poly(A)-tail length during oocyte maturation, with relevance to human fertility
Source: Nat Commun. 2025 Aug 1;16:7079. doi: 10.1038/s41467-025-62171-5 (PMC12316995; doi:10.1038/s41467-025-62171-5)
Supplement: Supplementary file 1 — Supplementary Information [file 41467_2025_62171_MOESM1_ESM.docx]

**Supplementary Information**

**
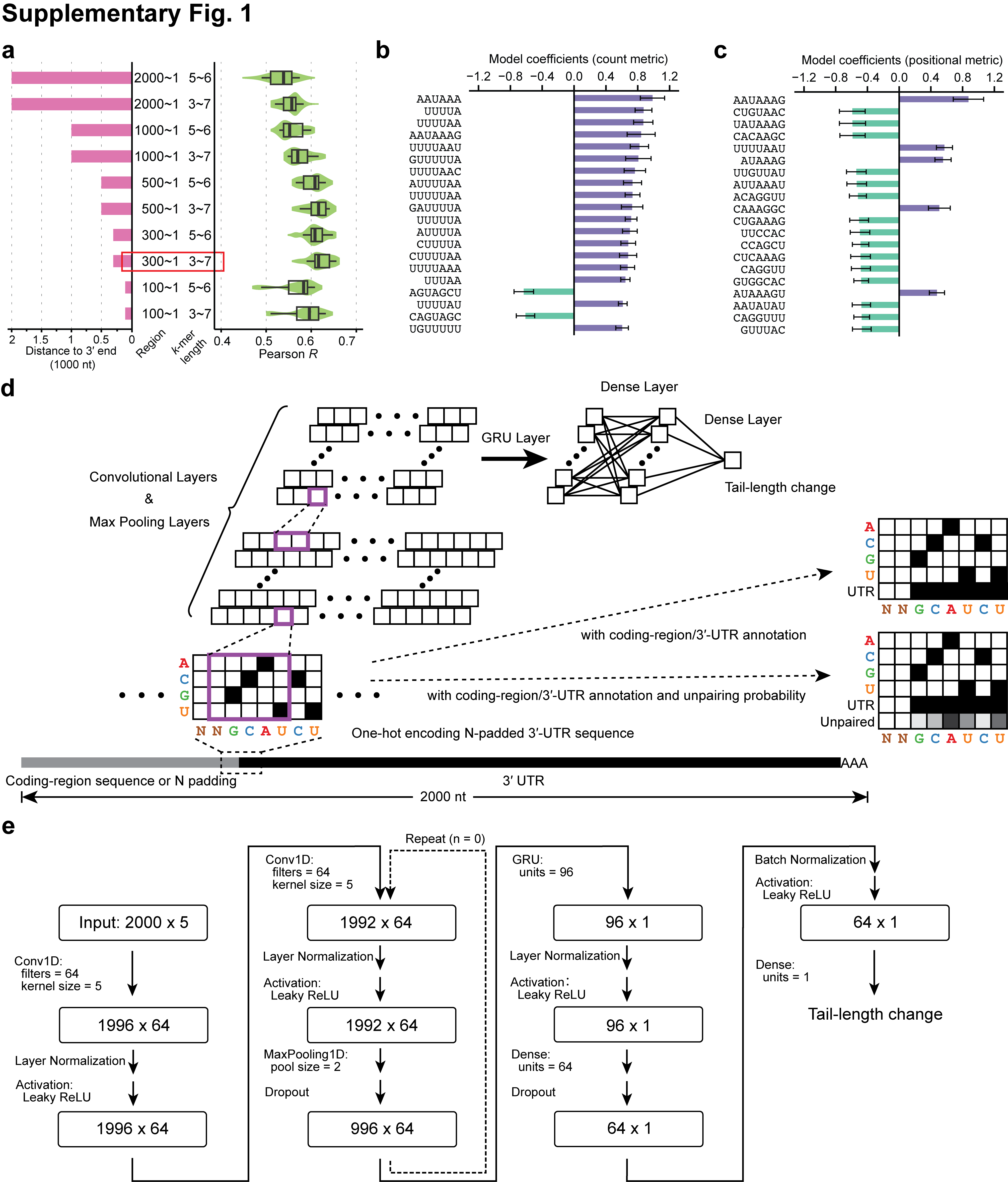
Supplementary Fig. 1: Additional data supporting the prediction of tail-length changes by multiple linear regression models and the architecture of PAL-AI.**

**a** Prediction performance of the multiple linear regression models trained on different input regions of mRNA and configured with different lengths of *k*-mers; otherwise, as in **Fig. 1f**. **b** Top-ranked features as the number of *k*-mers from the best-performing multiple linear regression models. Plotted are mean coefficients of the 20 top-ranked (by absolute value) features as the number of *k*-mers from the model. Error bars indicate standard deviation of coefficients of the 50 models from 10-fold cross-validation. **c** Top-ranked features as the positional metric of *k*-mers from the best-performing multiple linear regression models. As in (**b**), except plotted are mean coefficients of the 20 top-ranked (by absolute value) features as the positional metric (sum of the inverse of the distance between each *k*-mer and the 3' end) of *k*-mers from the model. **d** The architecture of PAL-AI for predicting tail-length changes. **e** Parameters of the architecture of PAL-AI. Shown for each layer are the name and parameters of the layer, and in boxes are the dimensions of the input and output matrices. The dashed arrow indicates repeats of the convolutional module. Different numbers of repeats were tested during hyperparameter searches.

**
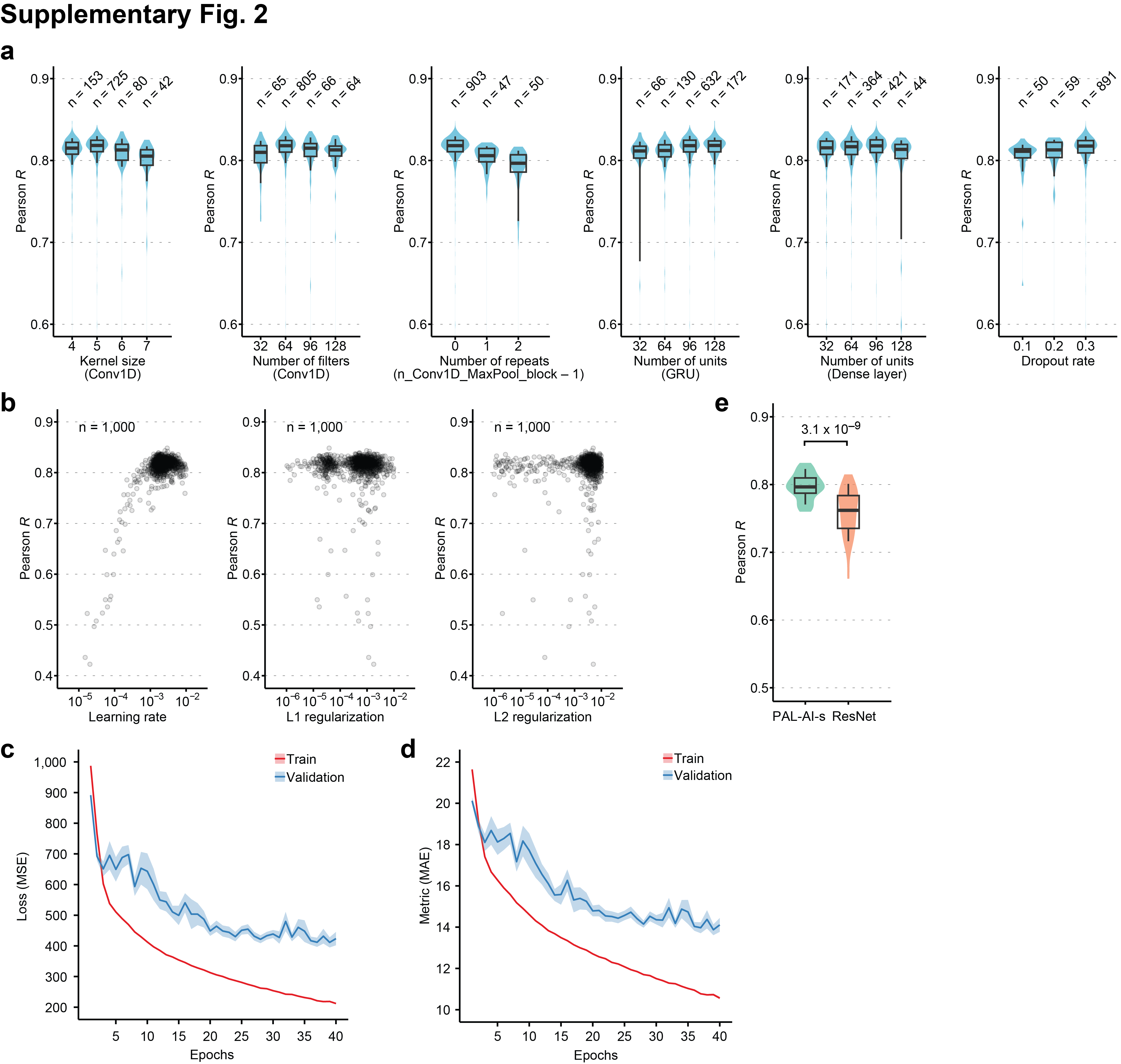
Supplementary Fig. 2:** **Hyperparameter optimization reveals optimal configuration parameters for PAL-AI.**

**a** Impact of discrete hyperparameters on PAL-AI performance. Shown are distributions of *R*_p_ values evaluating the correspondence between predicted and measured tail-length changes for different hyperparameter values in validation data. Numbers at the top indicate sample (trial) sizes for each condition. Box and whiskers indicate the 10th, 25th, 50th, 75th, and 90th percentiles. **b** Relationship between continuous hyperparameters and PAL-AI performance. Scatter plots show *R*_p_ values as a function of learning rate (left), L1 regularization (middle), and L2 regularization (right). Each point represents an individual model with specific hyperparameter values. **c** Learning curves showing training and validation loss. Mean squared error (MSE) is plotted against training epochs for both training (red) and validation (blue) datasets. Results represent means from 50 models (5 repeats for each fold in 10-fold cross-validation). Shaded areas indicate standard error of the mean. **d** Performance metrics during PAL-AI model training. Mean absolute error (MAE) is plotted against training epochs for both training (red) and validation (blue) datasets, analyzed as in (**c**). **e** Comparison of model architectures. Shown are distributions of *R*_p_ values evaluating the correspondence between predicted and measured tail-length changes for PAL-AI-s or ResNet implementations (with input sequence length of 2000 nt, 3′ UTR only). Ten-fold cross-validation of each model was repeated five times, generating 50 *R*_p_ values. Box and whiskers indicate the 10th, 25th, 50th, 75th, and 90th percentiles. The *P* value above the bracket was from a one-sided t-test.

**
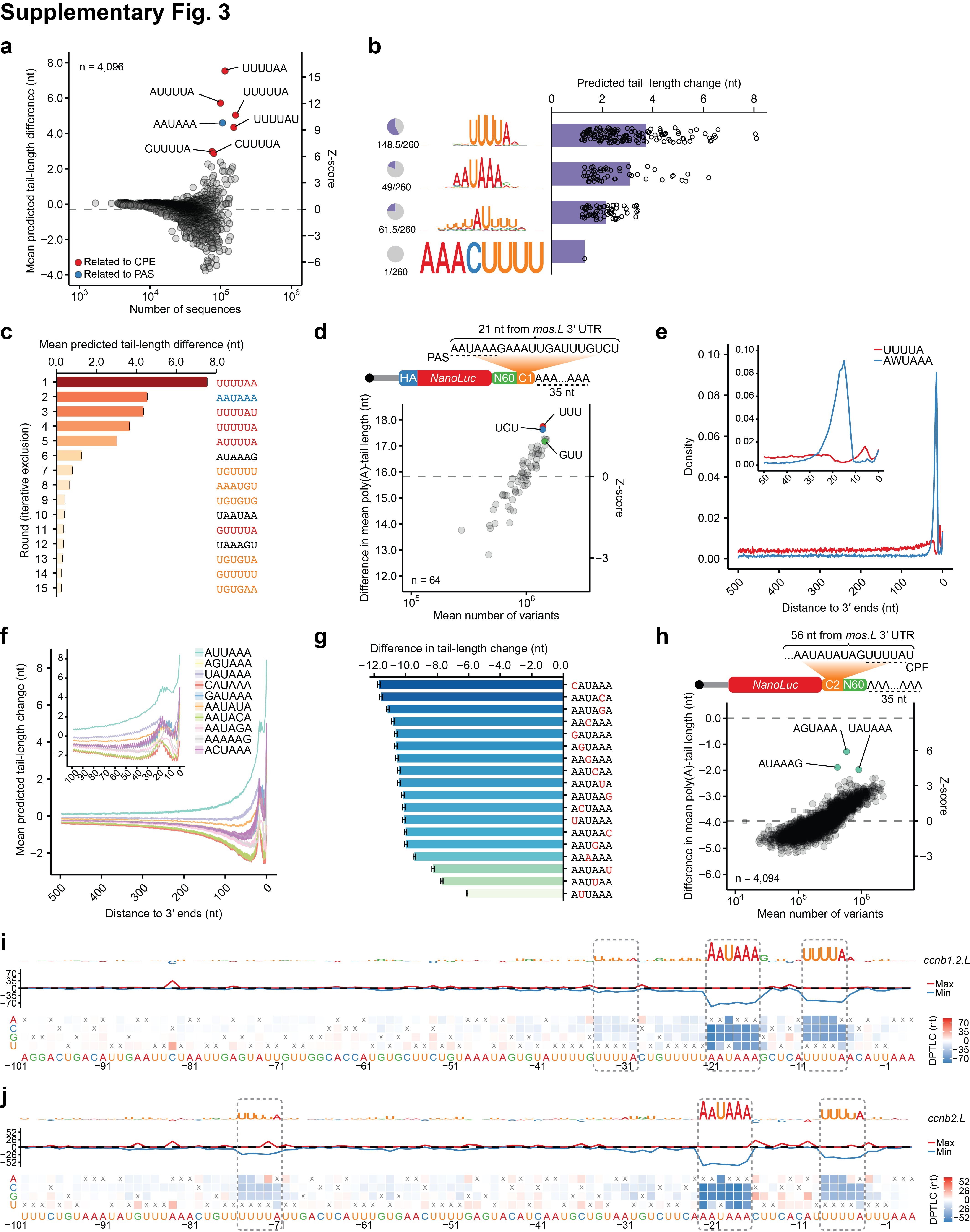
**

**Supplementary Fig. 3:** **Additional data supporting sequence elements and contextual features learned by PAL-AI.**

**a** As in **Fig. 2a**, except for consequences of gain of sequence motifs. **b** As in **Fig. 2b**, except for 8-mers most associated with the largest differences in predicted tail-length change between mutants in which 8-mers were gained and the wild-type. **c** As in **Fig. 2c**, except for top 6-mers associated with increased predicted tail-length change when gained in the in silico mutagenesis analysis. **d** Tail-length changes associated with each 3-mer within the 3' UTRs of the N60-PAS*^mos^* library^1^, comparing between 0 h and 7 h post-progesterone treatment. Plotted for each 3-mer are differences in mean tail lengths observed for mRNAs with that 3-mer. Only variants containing one CPE (UUUUA) were included in this analysis. **e** Densities of the PAS and the CPE along the last 500 nt of frog mRNA 3' UTRs. The inset shows the results for the last 50 nt of the 3' UTR. **f** As in **Fig. 2e**, except for PAS variants. All insertions that would disrupt an AAUAAA were excluded. **g** Reduced tail-length change for PAS variants. Shown are the mean differences in predicted tail-length change when replacing the canonical PAS (AAUAAA) with the indicated PAS variant. Nucleotides that differ from the canonical PAS are colored in red. Only variants within the last 300 nt of 3' UTRs were included in this analysis. Error bars: standard error of the mean. **h** Tail-length changes associated with each 6-mer within the 3' UTRs of the CPE*^mos^*-N60 library, comparing between 0 h and 5 h post-progesterone treatment^1^. Plotted for each 6-mer are differences in mean tail lengths observed for mRNAs with that 6-mer. Variants containing any canonical PAS (AAUAAA or AUUAAA) were excluded from this analysis. **i** Impact of single-nucleotide substitutions on predicted tail-length changes for the *ccnb1.2.L* mRNA 3' UTR; otherwise, as in **Fig. 2g**. **j** Impact of single-nucleotide substitutions on predicted tail-length changes for the *ccnb2.L* mRNA 3' UTR; otherwise, as in **Fig. 2g**.

**
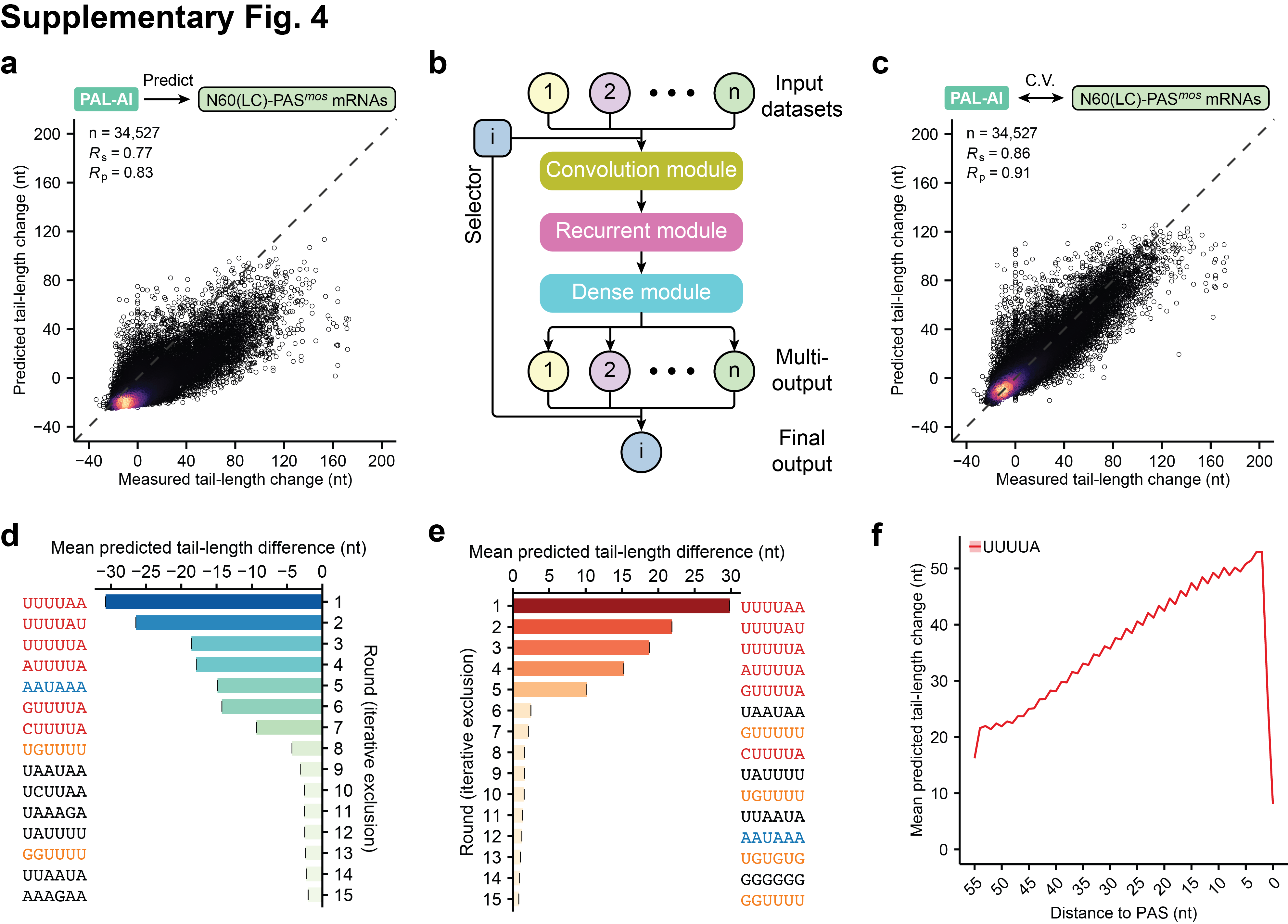
Supplementary Fig. 4:** **Additional data supporting the prediction of tail-length changes for synthetic mRNAs in frog oocytes.**

**a** Performance of the PAL-AI model trained on frog endogenous mRNAs when tested on injected mRNA reporters. Each point represents a variant in the N60(LC)-PAS*^mos^* library with at least 50 poly(A) tags in each of the two datasets, which were from oocytes 0 h and 7 h post-progesterone treatment; otherwise, as in **Fig. 1c**. **b** Diagram of the architecture of the multi-output PAL-AI model (PAL-AI-m). **c** Performance of a PAL-AI model trained on data generated from the injected N60(LC)-PAS*^mos^* mRNA library and tested using 10-fold cross-validation (C.V.). The 34,527 variants in the N60(LC)-PAS*^mos^* library with at least 50 poly(A) tags in each of the two datasets, which were from oocytes 0 h and 7 h post-progesterone treatment, were used in this analysis; otherwise, as in **Fig. 1c**. **d** Top 6-mers associated with decreased predicted tail-length change in the in silico mutagenesis analysis with a model trained on data generated from the injected N60(LC)-PAS*^mos^* mRNA library. Otherwise, this panel is as in **Fig. 2c**. **e** As in (**d**), except for top 6-mers associated with increased predicted tail-length change when gained in the in silico mutagenesis analysis. **e** Positional effects for the CPE predicted by a model trained on data generated from the injected N60(LC)-PAS*^mos^* mRNA library. Otherwise, this panel is as in **Fig. 2e**.


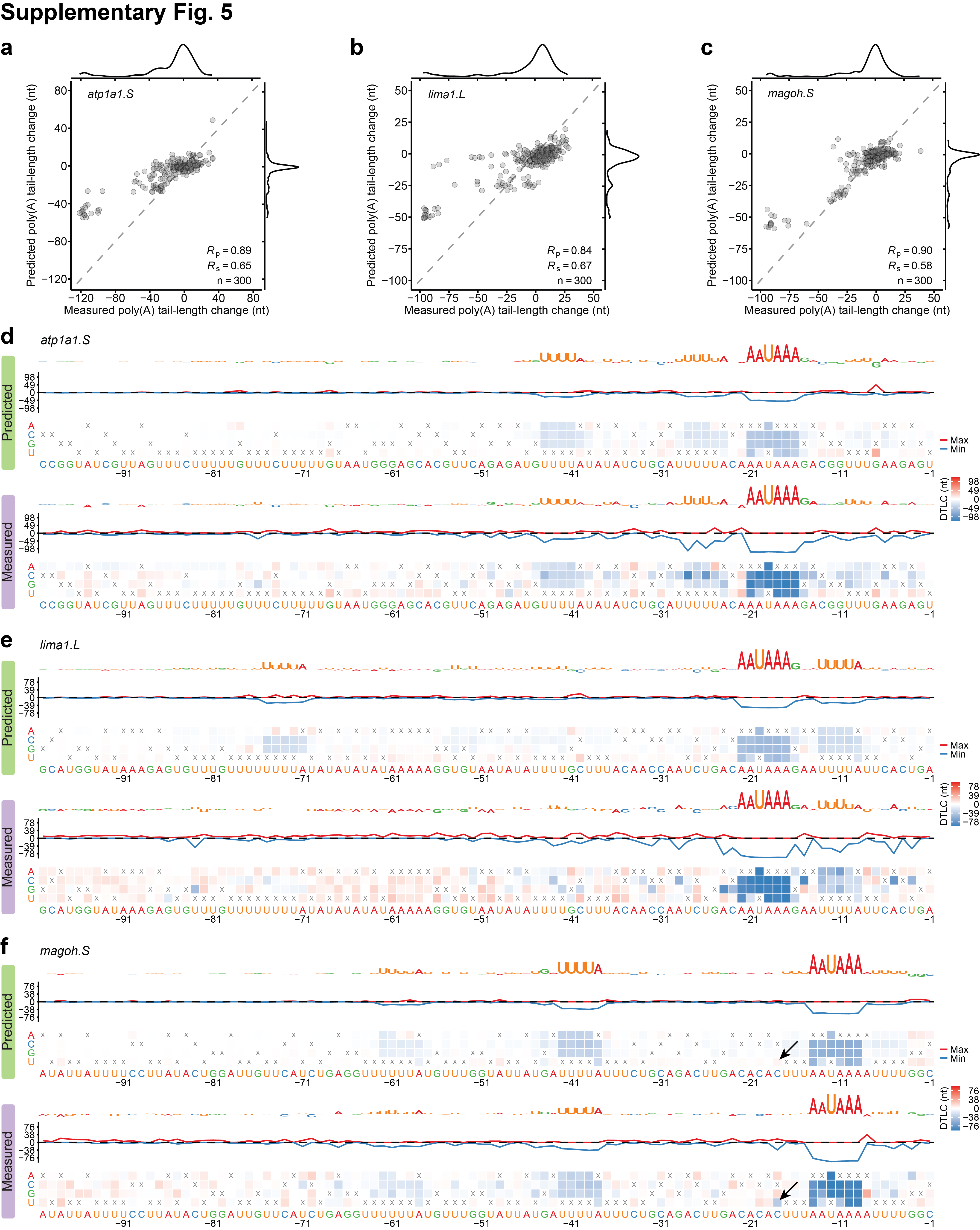


**Supplementary Fig. 5: Additional experimental validation of predicted effects of single-nucleotide substitutions.**

**a** PAL-AI prediction of the effects of single-nucleotide substitutions of the *atp1a1.S* mRNA. Otherwise, this panel is as in **Fig. 4d**. **b** As in (**a**), but for the *lima1.L* mRNA. **c** As in (**a**), but for the *magoh.S* mRNA. **d** Impact of single-nucleotide substitutions on predicted and measured tail-length changes for the *atp1a1.S* mRNA. Otherwise, this panel is as in **Fig. 4f**. **e** As in (**d**), but for the *lima1.L* mRNA. **f** As in (**d**), but for the *magoh.S* mRNA. Arrows indicate differences in predicted and measured tail-length changes resulting from a C-to-U substitution that creates a CPE overlapping with the PAS.

**
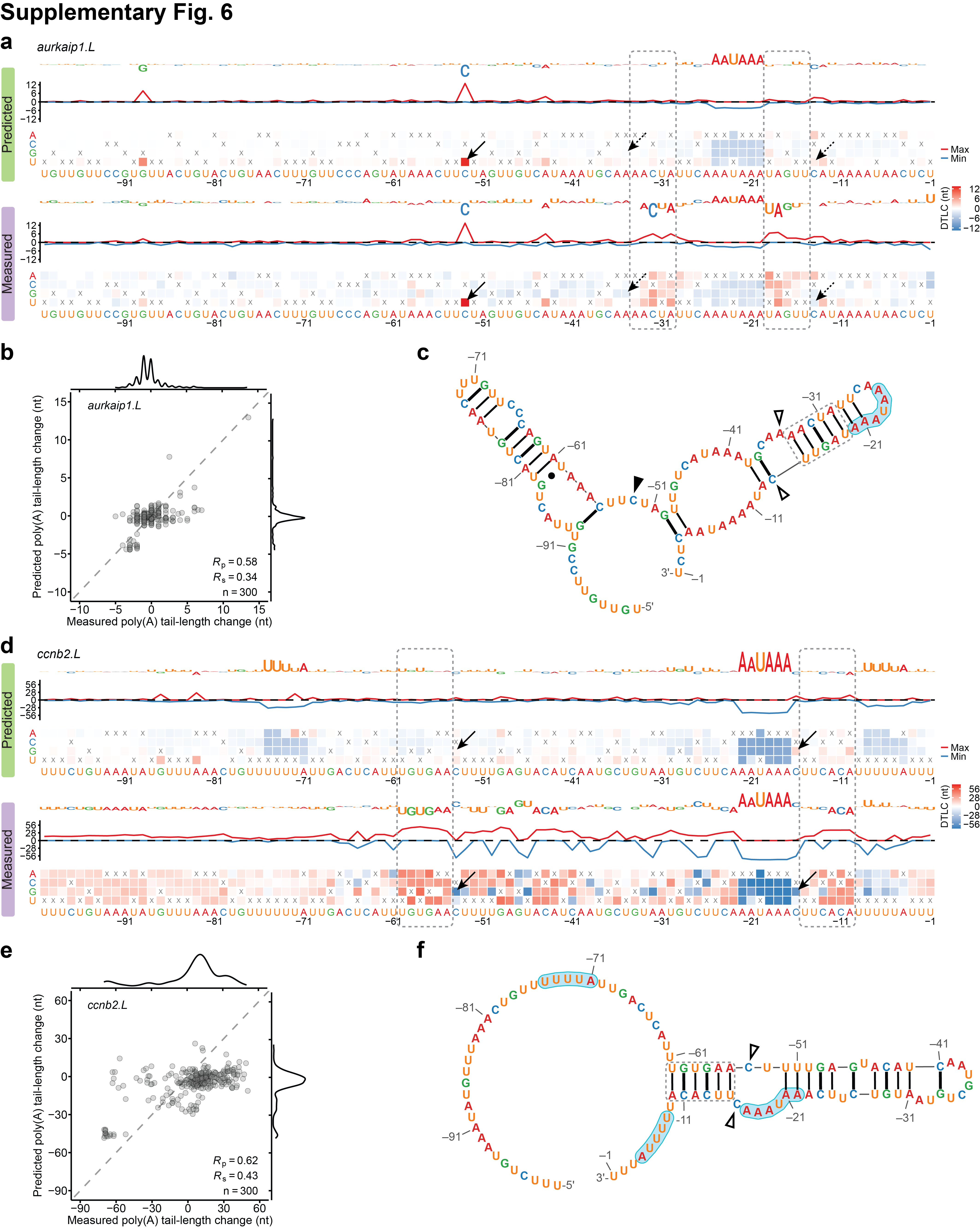
Supplementary Fig. 6: Additional analyses supporting tail-length changes affected by structural accessibility.**

**a** Impact of single-nucleotide substitutions on predicted and measured tail-length changes for the *aurkaip1.L* mRNA. Arrows indicate predicted and measured differences in tail-length changes of either a C-to-U substitution (solid) that creates a CPE or two substitutions (A-to-G at position –35 and C-to-U at position –14, dashed) that would increase the length of a predicted stem (dashed rectangles) expected to occlude the PAS. Otherwise, this panel is as in **Fig. 4f**. **b** PAL-AI prediction of the effects of single-nucleotide substitutions of the *aurkaip1.L* mRNA. Otherwise, this panel is as in **Fig. 4d**. **c** EternaFold-predicted maximum expected accuracy secondary structure of the last 100 nt of the *aurkaip1.L* 3' UTR. The dashed rectangle indicates the stem of a hairpin, as highlighted in (**a**). The triangles indicate positions of either a C-to-U substitution (solid) that creates a CPE in a predicted loop region with a tail-length change correctly predicted by PAL-AI or two substitutions (A-to-G at position –35 and C-to-U at position –14, hollow) that would increase the length of a predicted stem expected to occlude the PAS (shaded blue). **d** As in (**a**), but for the *ccnb2.L* mRNA. Arrows indicate predicted and measured differences in tail-length changes of C-to-G substitutions at positions –54 and –16, both of which create the potential for a C–G base pair that would increase the length of a predicted stem indicated with dashed rectangles. **e** As in (**b**), but for the *ccnb2.L* mRNA. **f** As in (**c**), but for the *ccnb2.L* mRNA. Shaded in blue are two CPEs and a PAS. The triangles indicate two positions where C-to-G substitutions would extend the predicted stem indicated with the dashed rectangle.

**
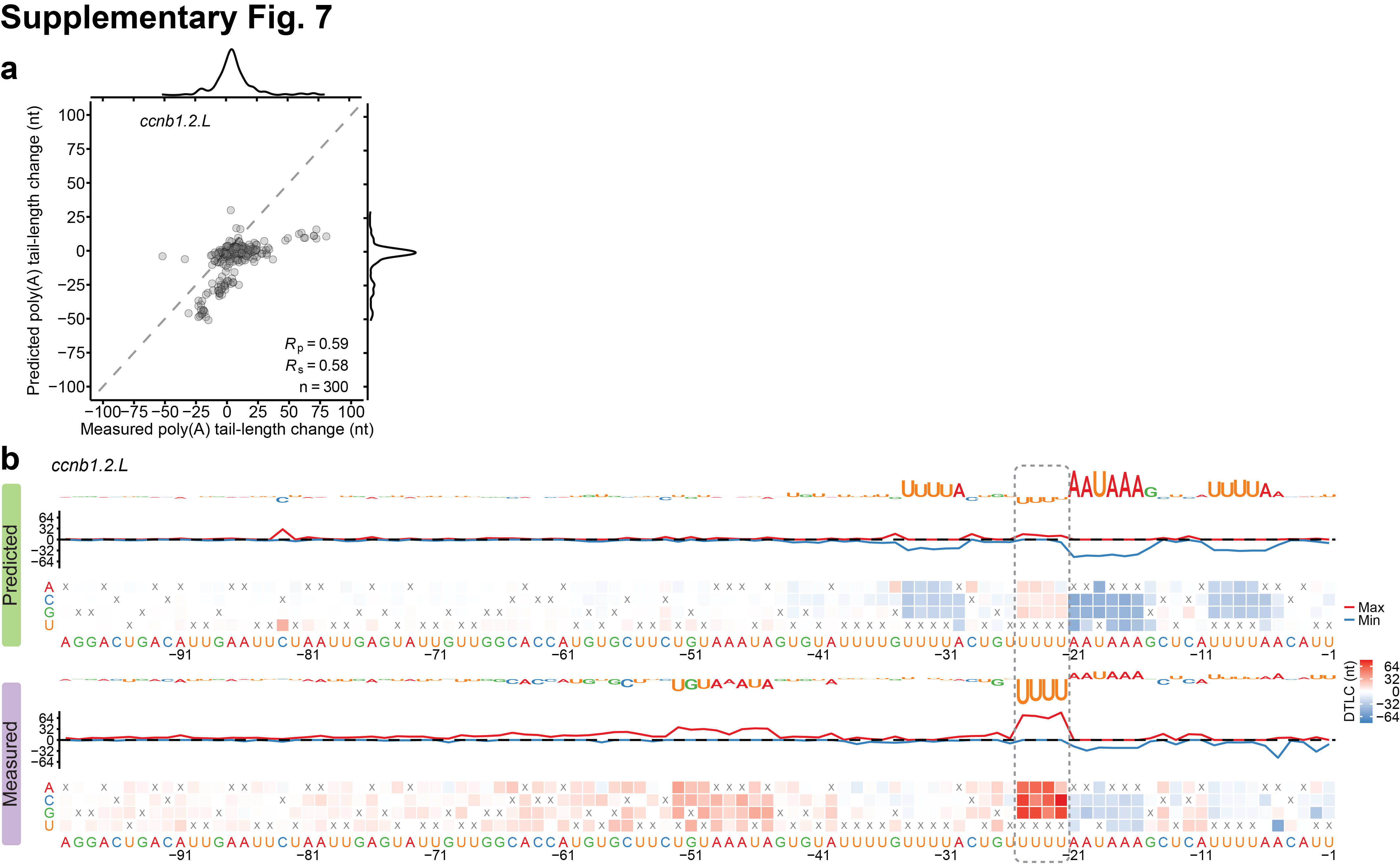
Supplementary Fig. 7: Additional experimental validation of predicted effects of single-nucleotide substitutions, exemplifying consequences of CPE-PAS overlap.**

**a** PAL-AI prediction of the effects of single-nucleotide substitutions of the *ccnb1.2.L* mRNA. Otherwise, this panel is as in **Fig. 4d**. **b** Impact of single-nucleotide substitutions on predicted and measured tail-length changes for the *ccnb1.2.L* mRNA. The dashed rectangle indicates a CPE that overlaps with a PAS. Otherwise, this panel is as in **Fig. 4f**.

**
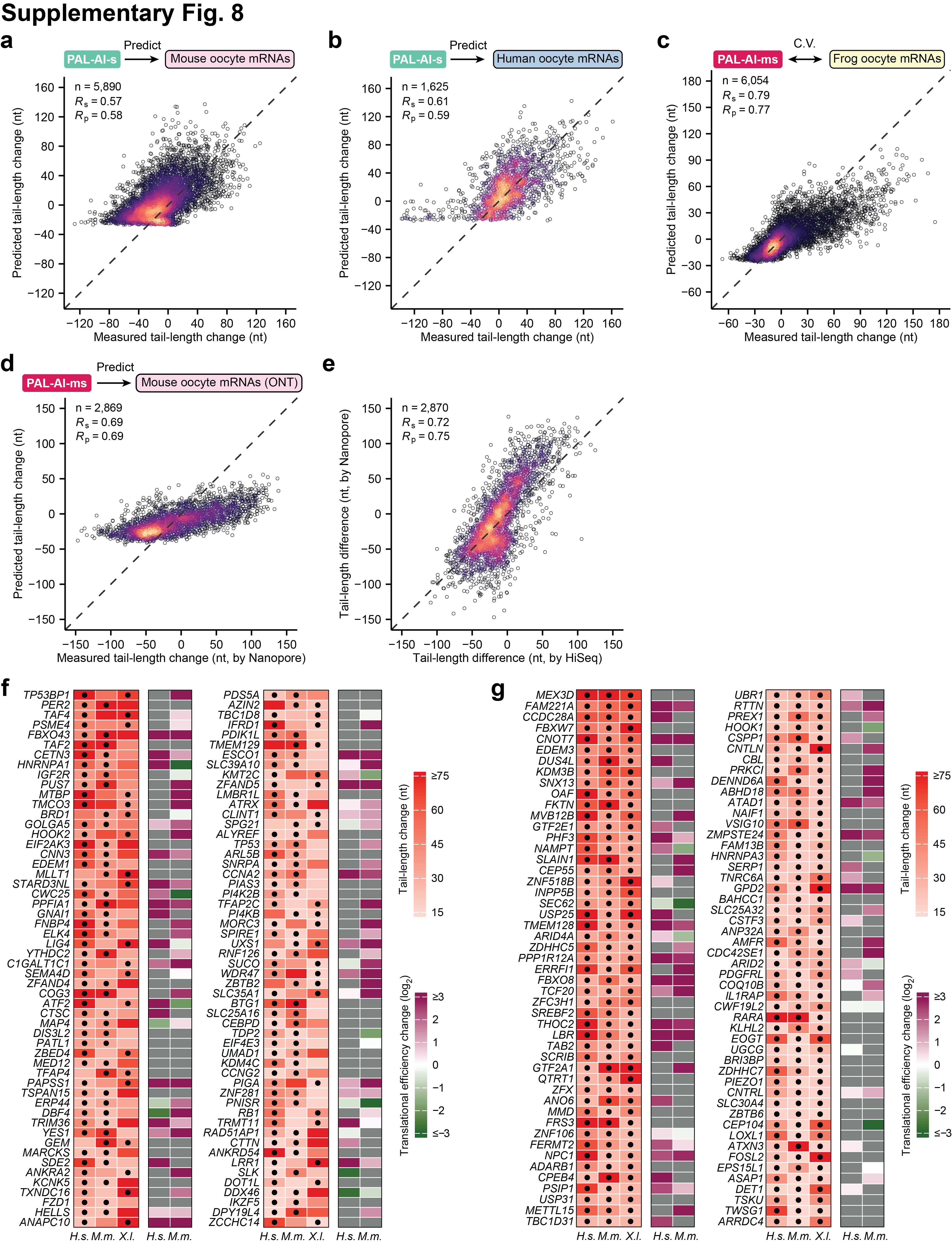
**

**Supplementary Fig. 8:** **Additional data supporting the prediction of tail-length change for endogenous mRNAs in mouse and human oocytes.**

**a** Performance of PAL-AI on mouse mRNAs. This panel is as in **Fig. 6a**, except that the predictions were made with the PAL-AI-s model trained solely on frog oocyte endogenous mRNAs. **b** Performance of PAL-AI on human mRNAs. This panel is as in **Fig. 6b**, except that the predictions were made with the PAL-AI-s model trained solely on frog oocyte endogenous mRNAs. **c** Performance of the PAL-AI-ms model on frog mRNAs. Otherwise, this panel is as in **Fig. 6a**. **d** Performance of the PAL-AI-ms model on mouse mRNAs with data generated from ONT in a published study^2^. This panel is as in Fig. 6a, except that the measured tail-length changes during mouse GV-to-MII oocyte maturation were obtained from ONT. **e** Correspondence between HiSeq and ONT measurements. Shown are tail-length differences measured either by HiSeq (x axis) or ONT (y axis) during mouse GV-to-MII oocyte maturation. Otherwise, this panel is as in **Fig. 1c**. **f** Genes predicted or shown to have substantial mRNA tail-lengthening. This panel is as in **Fig. 6d**, except genes with PAL-AI-predicted values in two of the three species are shown. **g** Genes predicted to have substantial mRNA tail-lengthening. This panel is in (**f**), except genes with PAL-AI-predicted values in all three species are shown.

**
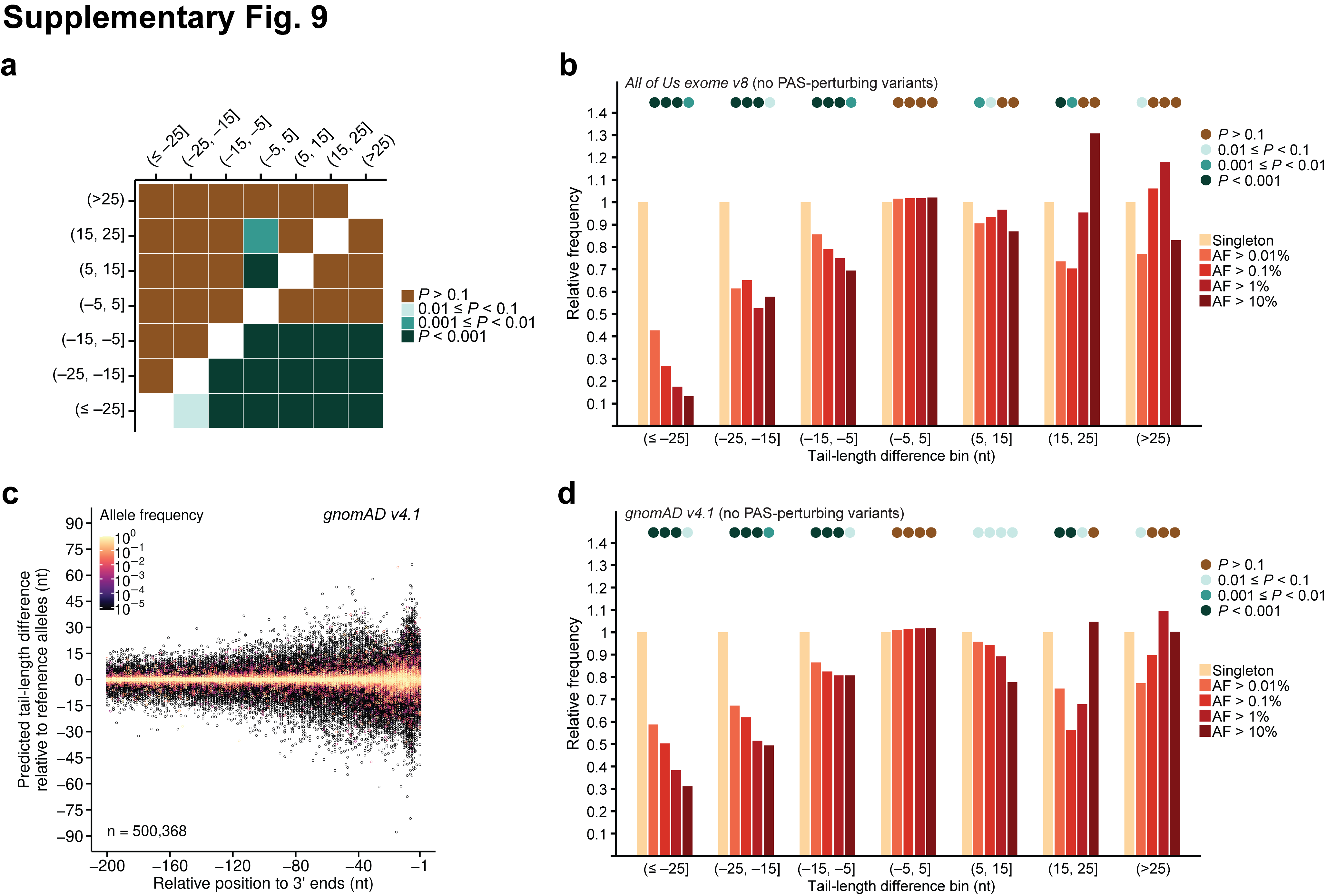
Supplementary Fig. 9: Additional data supporting negative selection of human variants predicted to disrupt poly(A)-tail lengthening.**

**a** Pairwise comparison of phyloP scores. Shown are binned *P* values from one-sided Mann–Whitney U tests of phyloP scores between groups with indicated differences in PAL-AI-predicted tail-length change, testing the alternative hypothesis that the mean phyloP scores of the group indicated on the y axis is greater than that indicated on the x axis. Exact *P* values are reported in **Supplementary Data 5**. **b** Depletion among frequent alleles predicted to disrupt poly(A)-tail lengthening, when excluding effects on PAS elements. This panel is as in **Fig. 7c**, except variants that introduced or eliminated PAS elements were excluded. **c** Analysis of gnomAD data, showing predicted effects on tail-length change for human variants. This panel is as in **Fig. 7b**, but for human variants in the gnomAD v4.1 dataset. **d** Analysis of gnomAD data, showing depletion among frequent alleles predicted to disrupt poly(A)-tail lengthening, when excluding effects on PAS elements. This panel is as in (**b**), but for human variants in the gnomAD v4.1 dataset.

**References**

1. Xiang, K., Ly, J. & Bartel, D. P. Control of poly(A)-tail length and translation in vertebrate oocytes and early embryos. *Dev. Cell* **59**, 1058-1074.e11 (2024).

2. Lee, K., Cho, K., Morey, R. & Cook-Andersen, H. An extended wave of global mRNA deadenylation sets up a switch in translation regulation across the mammalian oocyte-to-embryo transition. *Cell Rep.* **43**, 113710–113710 (2024).
