# Supplementary material for: PAL-AI reveals genetic determinants that control poly(A)-tail length during oocyte maturation, with relevance to human fertility
Source: Nat Commun. 2025 Aug 1;16:7079. doi: 10.1038/s41467-025-62171-5 (PMC12316995; doi:10.1038/s41467-025-62171-5)
Supplement: Supplementary file 2 — Description of Supplementary Information [file 41467_2025_62171_MOESM2_ESM.pdf]

Description of Additional Supplementary File

Supplementary Data 1: Parameters used in each PAL-AI model.

Supplementary Data 2: Oligo sequences.

Supplementary Data 3: Oligo sequences of the single-nucleotide mutagenesis library.

Supplementary Data 4: Sequences of the N60(LC)-PAS^{mos} library, the single-nucleotide mutagenesis library, and the tail-length standards.

Supplementary Data 5: Information on statistical tests.
