# Supplementary material for: PAL-AI reveals genetic determinants that control poly(A)-tail length during oocyte maturation, with relevance to human fertility
Source: Nat Commun. 2025 Aug 1;16:7079. doi: 10.1038/s41467-025-62171-5 (PMC12316995; doi:10.1038/s41467-025-62171-5)
Supplement: Supplementary file 8 — Reporting Summary [file 41467_2025_62171_MOESM8_ESM.pdf]

nature portfolio

Corresponding author(s): Kehui Xiang, David P. Bartel

Last updated by author(s): Jun 26, 2025

Reporting Summary

Nature Portfolio wishes to improve the reproducibility of the work that we publish. This form provides structure for consistency and transparency in reporting. For further information on Nature Portfolio policies, see our Editorial Policies and the Editorial Policy Checklist.

Statistics

For all statistical analyses, confirm that the following items are present in the figure legend, table legend, main text, or Methods section.

n/a	Confirmed	
☐	☒	The exact sample size (*n*) for each experimental group/condition, given as a discrete number and unit of measurement
☒	☐	A statement on whether measurements were taken from distinct samples or whether the same sample was measured repeatedly
☐	☒	The statistical test(s) used AND whether they are one- or two-sided<br>*Only common tests should be described solely by name; describe more complex techniques in the Methods section.*
☒	☐	A description of all covariates tested
☒	☐	A description of any assumptions or corrections, such as tests of normality and adjustment for multiple comparisons
☐	☒	A full description of the statistical parameters including central tendency (e.g. means) or other basic estimates (e.g. regression coefficient) AND variation (e.g. standard deviation) or associated estimates of uncertainty (e.g. confidence intervals)
☐	☒	For null hypothesis testing, the test statistic (e.g. *F*, *t*, *r*) with confidence intervals, effect sizes, degrees of freedom and *P* value noted<br>*Give P values as exact values whenever suitable.*
☒	☐	For Bayesian analysis, information on the choice of priors and Markov chain Monte Carlo settings
☒	☐	For hierarchical and complex designs, identification of the appropriate level for tests and full reporting of outcomes
☐	☒	Estimates of effect sizes (e.g. Cohen's *d*, Pearson's *r*), indicating how they were calculated

Our web collection on statistics for biologists contains articles on many of the points above.

Software and code

Policy information about availability of computer code

Data collection	Sequencing was performed on an Illumina HiSeq 2500
Data analysis	The code for PAL-AI is written in Python 3.8 and available at https://github.com/coffeebond/PAL-AI. Reporter mRNA tail-length sequencing data analyses were performed using a custom script written in Python 2.7 and available at https://github.com/coffeebond/MPRA_tail_seq.<br>The codes used to generate the figures are available at https://github.com/coffeebond/PAL-AI_paper.<br>The following softwares were used in this study:<br>Python (2.7 and 3.8)<br>R (4.1.0)<br>RNAfold (2.7.0)<br>UMICollapse (1.0.0)<br>STAR (2.7.1)<br>EternaFold (1.3.1)<br>RNAcanvas (Jan. 24, 2024 release)

For manuscripts utilizing custom algorithms or software that are central to the research but not yet described in published literature, software must be made available to editors and reviewers. We strongly encourage code deposition in a community repository (e.g. GitHub). See the Nature Portfolio guidelines for submitting code & software for further information.

Data

Policy information about availability of data

All manuscripts must include a data availability statement. This statement should provide the following information, where applicable:

- Accession codes, unique identifiers, or web links for publicly available datasets
- A description of any restrictions on data availability
- For clinical datasets or third party data, please ensure that the statement adheres to our policy

All standard sequencing data are available in the Gene Expression Omnibus under the accession number GSE280422. Raw intensity data for reporter mRNA tail-length sequencing cannot be deposited in public databases due to their large sizes and are available upon request. Other processed data are available at Zenodo with DOI: 10.5281/zenodo.15461000, except for that derived from gnomAD and the All of Us Research Program, due to privacy policies on human genetic information.
Oligo sequences used in this study are listed in Supplementary Data 2. Sequences of the oligo library used for the single-nucleotide mutagenesis library are listed in Supplementary Data 3. Sequences of the N60(LC)-PASmos library, the single-nucleotide mutagenesis library, and the tail-length standards are listed in Supplementary Data 4.
Other publicly available data analyzed in this study are indicated in the relevant sections of Methods. To access the genomic data reported in the All of Us Research Program, a Controlled Tier account is required on the Workbench (https://workbench.researchallofus.org).
Accession codes with links are listed below:
GSE280422 [https://www.ncbi.nlm.nih.gov/geo/query/acc.cgi?acc= GSE280422] (N60(LC)-PASmos library mRNA and single-nucleotide mutagenesis library mRNA tail-length data)
15461000 [https://doi.org/10.5281/zenodo.15461000] (Processed data in this study)
GSE228001 [https://www.ncbi.nlm.nih.gov/geo/query/acc.cgi?acc=GSE228001] (Mouse oocyte mRNA tail-length data measured by ONT)
GSE241107 [https://www.ncbi.nlm.nih.gov/geo/query/acc.cgi?acc=gse241107] (frog oocyte mRNA, N60-PASmos library mRNA, and CPEmos-N60 library mRNA tail-length data; reprocessed mouse and human oocyte translational efficiency data)
HRA001911 [https://ngdc.cncb.ac.cn/gsa-human/browse/HRA001911] (human oocyte tail-length data)
GSE197265 [https://www.ncbi.nlm.nih.gov/geo/query/acc.cgi?acc=GSE197265] (human oocyte ribosome-footprinting profiling and mRNA-seq data)
GSE165782 [https://www.ncbi.nlm.nih.gov/geo/query/acc.cgi?acc=GSE165782] (mouse oocyte ribosome-footprinting profiling and mRNA-seq data)
Fig. 4g [https://static-content.springer.com/esm/art%3A10.1038%2Fs41556-022-00928-6/MediaObjects/41556_2022_928_MOESM4_ESM.xlsx] (source data for GFP signals of the GFP-Snd1 3' UTR reporter presented in Fig. 6c)
gnomAD v4.1 [https://gnomad.broadinstitute.org/data] (human genomic variant data reported in gnomAD)
All of Us Research CDRv8 [https://workbench.researchallofus.org/] (human genomic variant data reported in All of Us Research Curated Data Repository Exome v8)

Research involving human participants, their data, or biological material

Policy information about studies with human participants or human data. See also policy information about sex, gender (identity/presentation), and sexual orientation and race, ethnicity and racism.

Reporting on sex and gender	Not applicable
Reporting on race, ethnicity, or other socially relevant groupings	Not applicable
Population characteristics	Not applicable
Recruitment	Not applicable
Ethics oversight	Not applicable

Note that full information on the approval of the study protocol must also be provided in the manuscript.

Field-specific reporting

Please select the one below that is the best fit for your research. If you are not sure, read the appropriate sections before making your selection.

☒ Life sciences ☐ Behavioural & social sciences ☐ Ecological, evolutionary & environmental sciences

For a reference copy of the document with all sections, see nature.com/documents/nr-reporting-summary-flat.pdf

Life sciences study design

All studies must disclose on these points even when the disclosure is negative.

Sample size	No sample size was pre-determined.
Data exclusions	During the HiSeq 2500 run for both the N60(LC)-PASmos and single-nucleotide mutagenesis libraries, a sequencing artifact occurred at cycle

Data exclusions	26 of read 1, resulting in a repeated base call at cycle 26 and 27. Consequently, base 27 was removed from all reads prior to downstream analysis.
Replication	Model training and testing were replicated for 5 times in each fold of 10-fold cross-validation.
Randomization	Not applicable.
Blinding	Samples were blindly barcoded, sequenced, decoded after data acquisition.

Reporting for specific materials, systems and methods

We require information from authors about some types of materials, experimental systems and methods used in many studies. Here, indicate whether each material, system or method listed is relevant to your study. If you are not sure if a list item applies to your research, read the appropriate section before selecting a response.

Materials & experimental systems

n/a	Involved in the study
☒	☐ Antibodies
☒	☐ Eukaryotic cell lines
☒	☐ Palaeontology and archaeology
☐	☒ Animals and other organisms
☒	☐ Clinical data
☒	☐ Dual use research of concern
☒	☐ Plants

Methods

n/a	Involved in the study
☒	☐ ChIP-seq
☒	☐ Flow cytometry
☒	☐ MRI-based neuroimaging

Animals and other research organisms

Policy information about studies involving animals; ARRIVE guidelines recommended for reporting animal research, and Sex and Gender in Research

Laboratory animals	Xenopus laevis
Wild animals	No wild animals were used.
Reporting on sex	Not applicable.
Field-collected samples	Not applicable.
Ethics oversight	Animal experiments performed in this study were approved by the Massachusetts Institute of Technology Committee on Animal Care under protocol number 2306000544.

Note that full information on the approval of the study protocol must also be provided in the manuscript.

Plants

Seed stocks	Not applicable.
Novel plant genotypes	Not applicable.
Authentication	Not applicable.
